# Characterization of Plasma-Derived Extracellular Vesicles Isolated by Different Methods: A Comparison Study

**DOI:** 10.3390/bioengineering6010008

**Published:** 2019-01-17

**Authors:** Esther Serrano-Pertierra, Myriam Oliveira-Rodríguez, Montserrat Rivas, Pedro Oliva, Javier Villafani, Ana Navarro, M. Carmen Blanco-López, Eva Cernuda-Morollón

**Affiliations:** 1 Department of Chemical and Enviromental Engineering, Faculty of Chemistry, University of Oviedo, 33006 Oviedo, Spain; 2 Department of Analytical and Physical Chemistry, Faculty of Chemistry, University of Oviedo, 33006 Oviedo, Spain; oliveiramyriam@outlook.es; 3 Department of Physics, Gijón Polytechnic School of Engineering, University of Oviedo, 33006 Oviedo, Spain; rivas@uniovi.es; 4 Neurology Department, Hospital Universitario Central de Asturias, 33011 Oviedo, Spain; simplementepon@gmail.com (P.O.); jvillafani@yahoo.com (J.V.); e.cernudamorollon@yahoo.es (E.C.-M.); 5 Department of Morphology and Cellular Biology, Instituto de Neurociencias del Principado de Asturias (INEUROPA), University of Oviedo, 33006 Oviedo, Spain; anavarro@uniovi.es

**Keywords:** extracellular vesicles, enrichment, ultracentrifugation, nanoparticle tracking analysis, lateral flow immunoassay

## Abstract

Extracellular vesicles (EV) are small membrane structures released by cells that act as potent mediators of intercellular communication. The study of EV biology is important, not only to strengthen our knowledge of their physiological roles, but also to better understand their involvement in several diseases. In the field of biomedicine they have been studied as a novel source of biomarkers and drug delivery vehicles. The most commonly used method for EV enrichment in crude pellet involves serial centrifugation and ultracentrifugation. Recently, different protocols and techniques have been developed to isolate EV that imply less time and greater purification. Here we carry out a comparative analysis of three methods to enrich EV from plasma of healthy controls: ultracentrifugation, ExoQuick^TM^ precipitation solution (System Biosciences), and Total Exosome Isolation kit (Invitrogen). Our results show that commercial precipitation reagents are more efficient and enable higher EV enrichment factors compared with traditional ultracentrifugation, although subsequent imaging analysis is not possible with some of them. We hope that this work will contribute to the current research on isolation techniques to assist the progress of clinical applications with diagnostic or therapeutic objectives.

## 1. Introduction

Extracellular vesicles (EV) are small membrane-bound particles (less than 1 µm in diameter) containing proteins, lipids and nucleic acids from donor cells that may be functional in recipient cells. Recently EV have emerged as means of communication between distant cells. They are produced by several mechanisms: by fusion of multivesicular bodies and the plasma membrane (usually referred as exosomes), or directly by plasma membrane budding in response to intracellular or extracellular stimuli [1,2,3]. In the last years, it has been shown that EV secretion and EV-mediated pathways are important, not only in normal biological processes, but also playing a relevant role in several diseases. Thus, EV have a great potential in biomedical applications, including their use as novel theranostic tools.

A proper and standardized method of isolation and characterization of EV is required to deepen our biological understanding of EV and their potential use as biomarkers [4], or to explore their possibilities as drug delivery systems [5,6]. The source of EV (biological fluids or cell culture supernatants), the starting volumes, or the possibility to scale up or down according to the number of vesicles required for further analysis are, among others, factors to consider when choosing an EV isolation protocol. To date, the most widely used method for EV purification is differential centrifugation at increasing speeds. The main drawback of this method is that it is time consuming, especially when isolating EV from biological fluids. EV can also be isolated by size-exclusion chromatography, which has been shown to significantly reduce albumin contamination in plasma-derived EV compared with the ultracentrifugation method [7]. Immunoaffinity-based methods are useful for the isolation of EV from specific cell types [8,9]. More recently, several commercial kits have been developed to precipitate EV at low centrifugation speeds, and with these techniques exosomal RNA of good quality can be extracted [10]. In general, commercial kits take shorter times and require less starting volume (in the case of biological fluids). From the variety of methods to isolate EV currently available (reviewed by Li et al. [11]), the choice of the most suitable one, taking into account subsequent EV characterization and analysis, is crucial. 

As mentioned, EV are considered potential biomarkers for diagnosis and prognosis of several diseases such as colorectal cancer [12], prostate cancer [13], glioblastoma [14], or cerebrovascular disease [15]. The study of their protein or nucleic acid content is also promising for the diagnosis of prognosis of cardiovascualar disease [16] or multiple myeloma [17]. Taken together, plasma from peripheral blood is an excellent source of EV as it is easily collected and does not usually cause discomfort to the patient. Maybe the major disadvantage of this biological fluid is that it contains abundant soluble proteins and aggregates, which may interfere with the isolation method. Nevertheless, plasma-derived EV are potential, noninvasive biomedical tools, and the development of portable analytical platforms to detect them is in progress [18,19]. Short real-time detection and sensitivity in bioanalysis are the major challenges to address in this field, but optimal methods for the enrichment of EV are essential before target detection.

In this work, a comparative study of three different procedures to enrich EV from plasma of healthy donors has been carried out. Although the gold standard method to isolate EV is differential centrifugation, the short times provided by precipitation kits could be attractive for clinicians that would need to take a medical decision based on EV content in blood. Even though these methods do not totally avoid free protein contamination, we have previously shown that rapid in vitro tests based on lateral flow immunoassays for EVs could be combined with these isolation kits [20]. A quantitative data for EVs could be taken in a couple of hours, with a limit of detection comparable with other instrumental analysis techniques. However, there could be concerns on how the precipitation agents or the high rotation speeds affect the properties of the EVs and the efficiency of the immunochromatographic strips. With the aim of assessing how significant these effects were, we have evaluated the efficiency of ultracentrifugation and two commercial kits (Total Exosome Isolation kit from Invitrogen and ExoQuick). The isolated EV were characterized and the suitability of the three methods to enable observation of the isolated vesicles by transmission electron microscopy (TEM) and detection by lateral flow immunoassay (LFIA) was checked.

## 2. Materials and Methods

### 2.1. Plasma Samples

Blood samples from healthy controls (3 males, age range 41–48) were collected after obtaining written informed consent to the study, which was approved by the Ethics Committee of Hospital Universitario Central de Asturias, and conforms with the principles outlined in the Declaration of Helsinki. Peripheral venous blood was collected in Vacutainer (Becton Dickinson) tubes with EDTA as anticoagulant and processed within 30 min of collection. Blood was first centrifuged for 30 min at 1550 g to remove cells. Platelet-free plasma (PFP) was obtained by centrifugation for 30 min at 3200 g. Aliquots of plasma were maintained at −80 °C until use or further centrifuged to isolate extracellular vesicles.

### 2.2. Enrichment of Extracellular Vesicles

#### 2.2.1. Ultracentrifugation 

PFP was processed by successive centrifugations at increasing speeds. It was first centrifuged for 30 min at 11,000× *g*. Supernatant was recovered and further centrifuged at 18,000× *g* for 30 min. The final supernatant was ultracentrifuged for 2 h at 100,000× *g*, then the pellet washed in PBS and centrifuged again for 2 h at 100,000× *g*.

#### 2.2.2. EV Enrichment Based on Precipitation Reagents 

EV were purified using the ExoQuick^TM^ precipitation solution (System Biosciences, Palo Alto, CA) or the Total Exosome Isolation kit (Invitrogen, Carlsbad, CA), according to the manufacturer’s instructions. 

**Figure 1:**
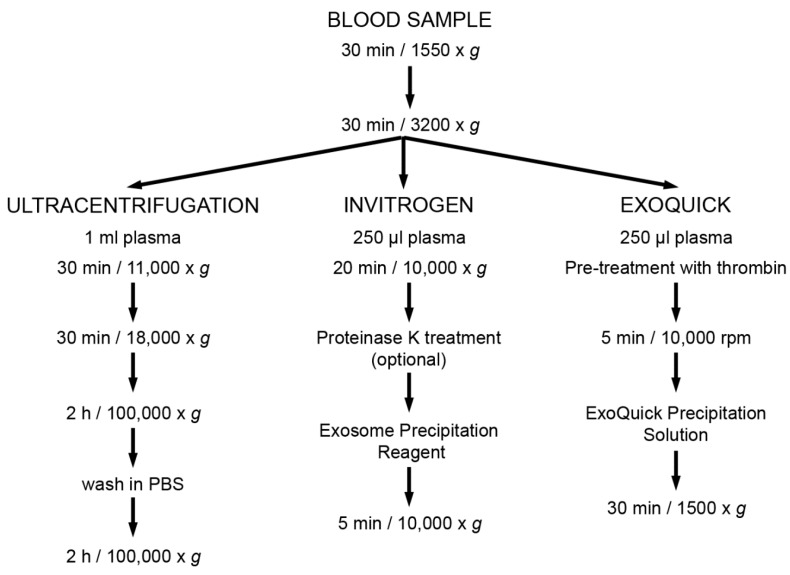
Methods of extracellular vesicles (EV) enrichment. Summary of the main steps of each method for enrichment of plasma-derived EV.

The main steps of these techniques are shown in Figure 1.

### 2.3. Assessment of Total Protein Content 

The EV fractions were homogenized with 1× RIPA buffer (25 mM Tris pH 7.4, 150 mM NaCl, 1% NP-40, 1% Na-Deoxycholate, 0.1% SDS). In the case of EV isolated using Invitrogen kit with proteinase K digestion, proteins were precipitated with trichloroacetic acid (20% final concentration), and precipitates were homogenized with 1× RIPA buffer. Protein concentration was measured by bicinchoninic acid (BCA) assay kit (Thermo Scientific, Waltham, MA, USA) and the global protein content analyzed by SDS-PAGE and Coomassie blue staining. Equal volume or equal protein amount of each sample, mix with reducing Laemmli-buffer (LB) was loaded.

### 2.4. Western Blot 

Equal volume or equal protein amount of each sample was mixed with reducing LB and separated in SDS-PAGE. Proteins were transferred to PVDF membrane (Amersham, GE Healthcare, Munich, Germany). Membranes were blocked in 5% non-fat milk in TBS-T for 2 h at room temperature and then incubated with rabbit polyclonal anti-CD63 (Santa Cruz Biotech; Santa Cruz, CA, USA) overnight at 4 °C with gently rocking. Membranes were washed and HRP-conjugated antibodies were added for 1 h at room temperature. Blots were developed with the ECL detection system. Secondary antibodies HRP-conjugated were from Dako (Glostrup, Denmark). 

### 2.5. Dynamic Light Scattering (DLS) and Nanoparticle Tracking Analysis (NTA) 

Size distribution of EV was measured by DLS using a Zetasizer Nano ZS ZEN3600 (Malvern Instruments, Malvern, UK) equipped with a solid-state He-Ne laser at 633 nm wavelength. The intensity of the scattered light was measured at 173°. All measurements were undertaken in triplicates at 25 °C. Data processing and analysis were performed using Zetasizer software version 7.03.

Concentration and size distribution of the isolated EV were determined using a NanoSight LM10 instrument (Malvern, Worcestershire, UK) and NTA 3.1 software at Nanovex Biotechnologies S.L (Asturias, Spain). Samples were diluted 1:1000–1:5000 in 10 mM HEPES 7.4 to achieve a particle concentration ranging from 10^6^ to 10^9^ particles/mL. Three runs were recorded for each sample.

### 2.6. Transmission Electron Microscopy (TEM) 

EV fractions were diluted 1:10 in PBS, applied onto a 200-mesh carbon-coated nickel grid and washed in by sterile H_2_O. The nickel grid was negatively stained for 1 minute with 2% uranyl acetate and visualized using a JEOL 1011 electron microscope (JEOL Ltd., Tokyo, Japan) operated at 80 kV.

### 2.7. Lateral Flow Immunoassay (LFIA) 

Detection of purified EV by multi-targeted LFIA was performed as previously described [20]. Briefly, EV samples were homogenized with the detection antibody anti-CD63 (clone Tea 3/18) conjugated to 40 nm gold nanoparticles (AuNP-anti-CD63). As capture antibodies, anti-CD9 (clone VJ1/20) and anti-CD81 (clone 5A6) immobilized on the strip by an Isoflow dispensing system (Imagene Technology, USA) were used (dispense rate: 0.100 µL/mm). The immunostrip was then dipped and the samples allowed to run for 15 min in capillary flow through the strip. EV in the sample were sandwiched between the anti-tetraspanin immobilized on the strip and AuNP-anti-CD63. Unbound AuNP-conjugated migrated further to be captured by an anti-IgG antibody printed in the control line, which was used as system functional verification. An ESEQuant LR3 lateral flow strip reader (Qiagen, Madrid, Spain) was used to measure the signal intensities (in mV) and estimate the peak area of the signal (in mV × mm).

### 2.8. Statistical Analysis

Statistical analysis was carried out with two-tailed Student’s *t*-test for methods comparison. Statistical significance was set at *p* < 0.05. Graphs show the mean + standard deviation (SD) of three independent experiments. Data were analyzed using R version 2.13 (www.r-project.org).

## 3. Results

### 3.1. Yield of Extracellular Vesicles Enrichment from Plasma by Ultracentrifugation and Precipitation Reagents

**Figure 2:**
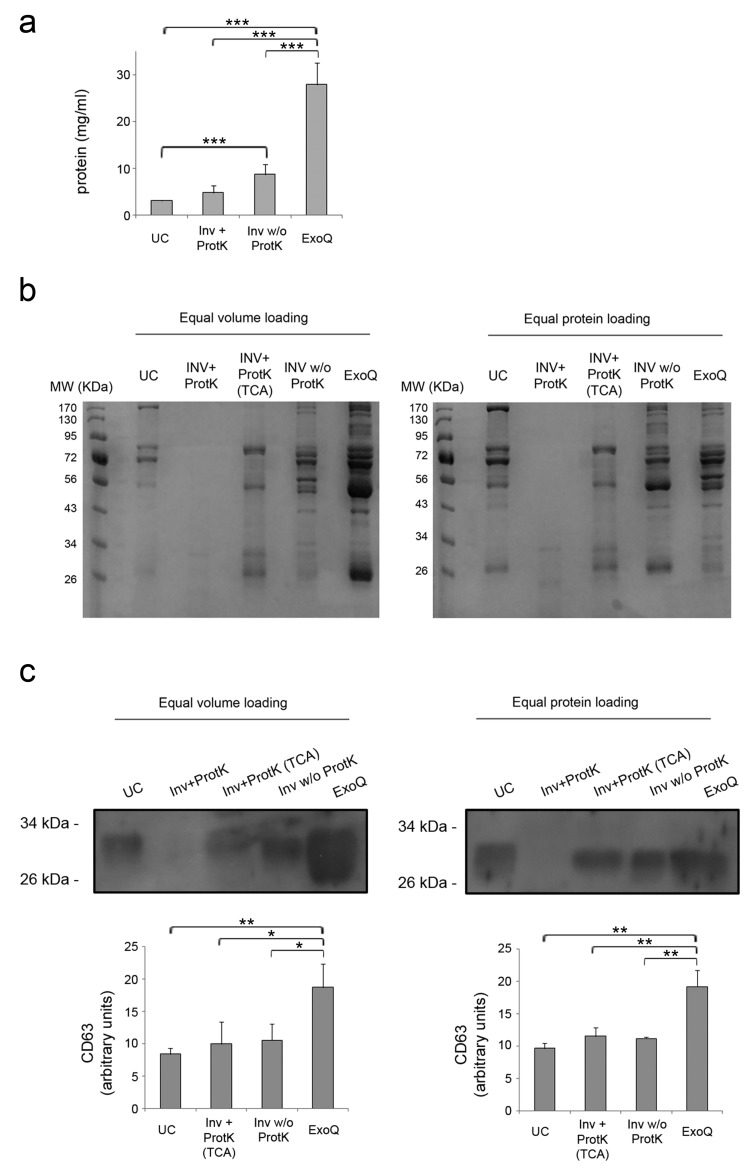
Efficiency of EV enrichment. (**a**) Protein concentration of the EV fractions isolated was determined by bicinchoninic acid (BCA) assay. The graph shows the mean + SD of the three independent experiments. (**b**) Coomassie blue staining of the EV fractions. Equal volume and equal amount of protein were loaded for a general protein stain. (**c**) Representative detection of CD63 in EV fractions by Western blot. Data shown are the mean + SD of three independent experiments. UC: Ultracentrifugation; INV+ProtK: Invitrogen kit and treatment with proteinase K; INV+ProtK (TCA): TCA precipitated protein from INV+ProtK fractions; INV w/o ProtK: Invitrogen kit without digestion with proteinase K; ExoQ: ExoQuick kit. * *p* < 0.05; ** *p* < 0.01; *** *p* < 0.001.

EV from 250 µL of plasma were isolated using Invitrogen and ExoQuick kits. In the case of Invitrogen kit, the isolation was performed with and without the optional step, which consists of a previous digestion of soluble proteins using proteinase K. For ultracentrifugation, the starting volume was scaled up to 1 mL of plasma, since protein levels were undetectable and therefore not suitable for subsequent Western blot analysis. The mean protein concentrations obtained from EV fractions and the p-values obtained in the statistical analysis are shown in Figure 2a. The ExoQuick method yielded the highest protein content (27.98 ± 4.66 mg/mL), followed by Invitrogen reagent (4.76 ± 2.09 mg/mL with proteinase K digestion, and 8.70 ± 1.55 mg/mL without this step). The amount of protein determined in EV fractions obtained by ultracentrifugation was the lowest one (3.05 ± 0.19 mg/mL) even though the starting volume of plasma was four times that used for enrichment of EV with commercial precipitation reagents. These results were confirmed when equal volumes of each sample (1 µL) were stained with Coomassie blue (Figure 2b). Surprisingly, we could not detect stained proteins of the EV fractions isolated using Invitrogen kit and proteinase K digestion, as recommended by the manufacturer. This was not observed neither when we skipped this step, nor when we precipitated protein with tricholoracetic acid (TCA) from EV fractions treated with proteinase K. We also stained the same amount of protein (10 µg) of each fraction and observed more variety of proteins when isolating EV using Invitrogen without proteinase K treatment and ExoQuick kit. Fractions that were precipitated with TCA showed less bands in comparison with EV fractions precipitated without digestion with proteinase K. Whether these absent bands corresponded to soluble proteins efficiently digested by Proteinase K or they were just a consequence of the TCA protein precipitation procedure cannot be ruled out. 

In order to approximate the overall efficiency of the different methods, equal volumes and equal amounts of protein from the different fractions were also analyzed by Western blot (Figure 2c). The highest levels of the tetraspanin CD63 were detected in EV fractions isolated using ExoQuick in comparison with Invitrogen (*p* < 0.05) and with ultracentrifugation (*p* < 0.01), showing that this is the most efficient method followed by Invitrogen kit. We found similar results when investigating the levels of CD63 in the EV isolated by loading equal amounts of protein, since they were found higher in fractions isolated with ExoQuick in comparison with the two other methods (*p* < 0.01). To evaluate levels of CD63 in fractions treated with proteinase K in Invitrogen kit it was necessary to precipitate the protein with TCA. According to the manufacturer’s instructions, treatment with proteinase K is optional, but we tested if addition of this enzyme affected the other downstream analyses and found no differences or issues in any other method employed. Therefore, we present our next results of Invitrogen isolation kit only with the treatment with proteinase K. 

### 3.2. Characterization of Plasma-Derived Extracellular Vesicles

**Figure 3:**
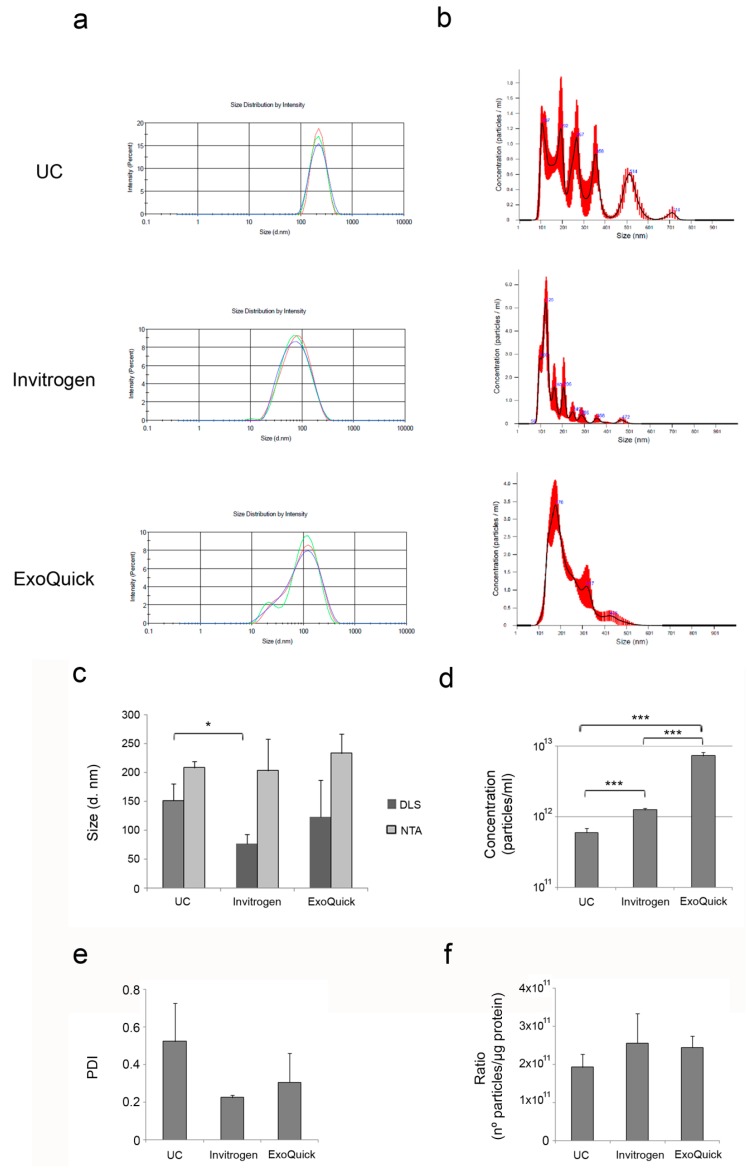
Characterization of plasma-derived EV. Hydrodynamic size distribution profiles of isolated EV measured by (**a**) DLS and (**b**) NTA. (**c**) Mean values + SD of the diameter sizes measured by DLS and NTA (n = 3). (**d**) Particle concentration of the EV fractions was measured by NTA. The graph shows the mean + SD of three independent experiments. (**e**) Mean values + SD of the PDI measured by DLS (n = 3). (**f**) Normalization of EV concentration determined by NTA per protein concentration measured by BCA. The graph shows the mean + SD of three independent experiments. * *p* < 0.05; ** *p* < 0.01; *** *p* < 0.001.

The size distribution of the isolated EV was obtained by dynamic light scattering (DLS) and nanoparticle tracking analysis (NTA) (Figure 3a,b). Both techniques are based on the measurement of Brownian motion relating it to the hydrodynamic diameter through the Stokes–Einstein equation [21,22]. However, DLS uses Rayleigh scattering for the estimation of the particle diameter (d). This intensity is proportional to d^6^. This means that 80 nm diameter will scatter 10^6^ (on million) times more light than the particles with smaller size, and therefore, the overall contribution of the smaller particles is overestimated with this technique. Given that EV fractions isolated from plasma contain a range of particle populations with different average sizes, NTA may be more suitable for this characterization, since DLS takes an overall signal while NTA works on a particle-by-particle basis. The results of the size of the isolated EV with both DLS and NTA are shown in Figure 3c for comparison. In general, NTA measures larger mean sizes of EV than DLS, as it detects the heterogeneity of EV size (range 95–438 nm). All the average sizes obtained from NTA measurements are similar and no significant differences were found but, as shown in Figure 3b, the EV population isolated by ultracentrifugation is much more heterogeneous than those isolated using the commercial kits. DLS measurements showed that EV isolated using commercial precipitation reagents (Invitrogen and ExoQuick) are smaller than those isolated by ultracentrifugation. 

Further analysis of the concentration of EV by NTA (Figure 3d) revealed that ExoQuick significantly isolates a larger number of particles per unit volume (7.383 × 10^12^ particles/mL), followed by Invitrogen kit (1.263 × 10^12^ particles/mL). Enrichment of EV by ultracentrifugation yielded the lowest concentration of EV (6.03 × 10^11^ particles/mL).

The polydispersity index (PDI) was also measured by DLS (Figure 3e) and the methods compared, although no significant differences were found. EV fractions isolated by ultracentrifugation obtained the highest PDI values, indicating more variable size of these vesicles in comparison with those isolated using the commercial kits. These results are in line with the NTA measurements, where a more heterogeneous population of EV was detected in the fractions isolated by ultracentrifugation.

In order to assess the EV purity, the ratio of particle number to protein concentration has been used [23,24]. Ratios over 3 × 10^10^ particles per microgram of protein are suggested to indicate high pure vesicle preparations [23]. According to this approach, all the methods produced highly pure fractions (Figure 3f), with no significant differences between the methods.

### 3.3. Electron Microscopy Observation and Detection of Extracellular Vesicles by LFIA 

**Figure 4:**
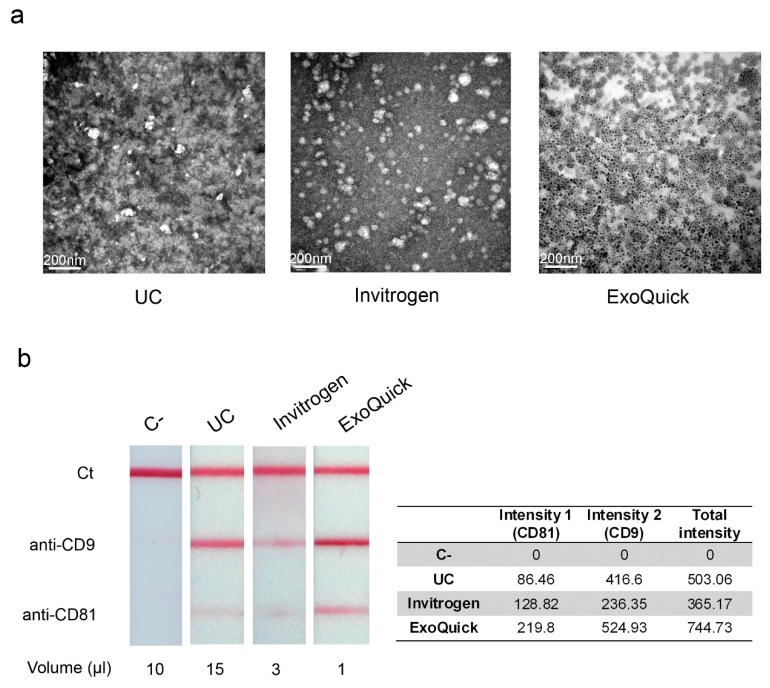
Observation and detection of isolated EV. (**a**) Transmission electron microscopy images representative of plasma-derived EV by ultracentrifugation or using commercial precipitation reagents (Invitrogen or ExoQuick). (**b**) Detection of the EV isolated by lateral flow immunoassay (LFIA), using anti-CD9 and anti-CD81 as capture antibodies, and reflectance measurements of AuNPs on each test line (estimated as the peak area of the signal in mV × mm). EV-depleted plasma was used as a negative control (C−). Unbound antiCD63-AuNP captured with anti-IgG were used as system functional verification (Ct).

After EV enrichment, we could observe intact EV by TEM. Figure 4a shows representative TEM images of EV isolated by ultracentrifugation, Invitrogen and ExoQuick kits. Equal volumes of each fraction were used. We can appreciate less EV per field in the sample isolated by ultracentrifugation, which is in agreement with the differences in the concentration of EV measured by NTA. Regarding the sample isolated using ExoQuick, the EV could not be observed due to the precipitation reagent of the kit, which interferes with the electron beam producing a blurred image. Fractions were filtrated in order to remove the precipitation reagent, but even so it was not possible to detect them. Therefore, we concluded that the ExoQuick kit is not suitable for TEM.

Multi-targeted LFIA were performed to enable a rapid on-site detection of EV. Anti-CD63 was used as detection antibody, anti-CD9 and anti-CD81 were the capture antibodies printed in two different test lines, and anti-IgG was used for the control line [25]. Figure 4b shows a representative example of the results obtained with the different methods to enrich EV and a negative-control sample obtained from EV-depleted plasma. Unbound antiCD63-AuNP captured with anti-IgG were used as system functional verification.

Evaluation of the optical signal intensities were performed by reflectance measurements. In agreement with our findings about particle concentration, the highest signal intensities were detected in the EV fractions isolated with ExoQuick, which required only 1 µL of sample. EV precipitated with Invitrogen kit were detected when applying 3 µL of the sample. However, it was necessary to scale up to 15 µL of EV isolated by ultracentrifugation to observe significant signal intensity.

## 4. Discussion

Current challenges in the study of the role of EV under physiological or pathological conditions lie with the standardization of protocols for EV purification. Different types of EV or EV from different sources (cell culture supernatants or biological fluids) require different enrichment or isolation approaches depending on the downstream studies. In addition, the free protein contamination in the case of biological fluids must be taken into account. Several procedures and technological solutions have been developed in the last years to satisfy the scientific community needs for the isolation of EV [26,27,28].

In this study, we have assessed the efficiency of three different methods of EV enrichment from plasma of healthy donors. We also studied the suitability of each method for further analyses, such as characterization of EV by DLS and NTA, and detection of EV by TEM or LFIA. Table 1 summarizes the main characteristics of each method. Overall, our BCA, Western blot, NTA and LFIA results correlate well and indicate that enrichment of plasma-derived EV using ExoQuick precipitation solution is more efficient than ultracentrifugation and the Invitrogen kit. 

In order to estimate the efficiency of the different methods we assessed the protein content (BCA), the detection of the exosomal marker CD63 by Western blot and the particle counting by NTA. These analyses indicated that ultracentrifugation is the least efficient method, whereas precipitation with ExoQuick reagent is the most efficient one. Invitrogen kit recommends digestion of soluble proteins present in plasma with proteinase K prior to EV precipitation. This, however, interferes with the eletrophoretic mobility of proteins and detection of the tetraspanin CD63 by Western blot. Differences in the electrophoretic protein mobilities were also noted in urinary EV isolated using the Invitrogen kit, which does not require proteinase K treatment [29]. For isolation of EV from plasma this step is optional, and skipping it allows using the EV fractions for subsequent SDS-PAGE and Western blot. We have also checked that precipitation of proteins with TCA was required in fractions where proteinase K digestion was used if further analysis of EV by SDS-PAGE and Western blot was necessary. Nevertheless, this implies an additional step and handling of the samples that is not required when isolating EV by ultracentrifugation or using ExoQuick precipitation reagent.

The three methods employed in this study are suitable for subsequent characterization of EV by DLS and NTA. The purity of the EV fractions was determined as the ratio of number of particles to protein concentration; the three methods obtained similar purity, with no significant differences between them. The mean size of the EV obtained by NTA is similar in the three methods. However, we could observe that EV isolated by ultracentrifugation were more heterogeneous in size. This could be due to EV aggregation during ultracentrifugation, as previously suggested [30]. Measurements of size distribution by DLS indicated that EV obtained by ultracentrifugation had greater mean size in comparison with the other methods. This can be related to the greater heterogeneity of size detected by NTA and the greater PDI values obtained by DLS. In addition, it has to be taken into consideration that DLS cannot accurately resolve heterogeneous mixtures and tends to overestimate the contribution of the larger particles at the intensity of the scattered light that is used for correlation with the particle diameter. Since the population of EV isolated from plasma is a polydisperse sample, NTA is a better choice than DLS, not only for size analysis, but also because it provides a quantitative estimation of the concentration. 

Transmission electron microscopy is the gold standard for determining the size and morphology of EV. However, the study of size distribution can be skewed by the selection of the fields in which the micrographs are taken and requires tedious sample preparation. In conclusion NTA is preferable to TEM, or DLS, as previously reported [31]. In this study, electron microscopy was carried out to assess the suitability of the three different methods for observation of the isolated EV. We found that, accordingly to DLS and NTA analysis, EV obtained from UC are more heterogeneous in terms of size when compared to those isolated using Invitrogen kit. It was not feasible to properly visualize EV isolated using ExoQuick, since the precipitation reagent interferes with the electron beam. Therefore EV from UC and Invitrogen can be used directly for TEM to gather information on the size and structure of EV, but not those isolated with ExoQuick. Conversely, the three methods are suitable for EV detection by multi-targeted LFIA. However, larger volumes of the fractions of EV isolated by ultracentrifugation were required.

Previous reports have carried out comparative studies to elucidate the most suitable method to isolate EV [7,29,32,33,34]. The traditional ultracentrifugation and density gradient ultracentrifugation methods and immunoaffinity capture methodology were compared isolating exosomes from a human cancer cell line. Although ultracentrifugation yielded the highest protein content, immunoaffinity capture showed more efficiency in exosome capture and exosome markers enrichment [32]. Ultracentrifugation was neither reproducible nor efficient in the enrichment of serum-derived exosomes in comparison with ExoQuick [33]. We described here that ExoQuick is much more efficient than traditional ultracentrifugation in the enrichment of plasma-derived EV, and in addition it showed very good reproducibility. It is possible that ultracentrifugation on a sucrose cushion employed by Caradec et al. [33] may be even less reproducible than ultracentrifugation without density gradient, but this has not been demonstrated. Size exclusion chromatography could represent a better choice than ultracentrifugation when isolating EV from plasma, since it avoids albumin contamination. However, improvements in this technique should be made in order to optimize the efficiency of the isolation [7]. ExoQuick precipitation of EV from ascites has proved to be the best method in terms of purity and quantity of exosomal protein and RNA when compared with ultracentrifugation, size exclusion chromatography, and anti-EpCAM DynaBeads from Invitrogen [34]. It should be noted that anti-EpCAM magnetic beads isolated a specific subpopulation of EV, and this must be taken into account when comparing the RNA and protein yield. Furthermore, the efficiency of different methods could vary with the source of EV. Urinary EV were isolated by ultracentrifugation, by extraction with biotinylated lectin, and using different commercial kits (ExoQuick-TC, Norgen, and Invitrogen). In this case, EV obtained from each method differed according to the levels of different protein markers that were evaluated, being the ExoQuick-TC method the least efficient [29]. 

In this study we focused on the enrichment of plasma-derived EV. Having techniques that allow the isolation of these vesicles in a short time and require small sample volumes is highly valuable for clinical purposes. Although ultracentrifugation is nowadays the most commonly used method to isolate EV, it does not accomplish these requirements. On the contrary, the commercial kits used in this work are shorter (about 2 h or less) while better performances are achieved with only 250 µL of plasma. Therefore, they constitute a good alternative to ultracentrifugation if they are going to be combined with rapid in-vitro tests.

It should be noted that all the methods employed in this study are general approaches to isolate all circulating EV populations. Here we described that ExoQuick enables purification of larger numbers of EV per volume in comparison with Invitrogen kit or the traditional ultracentrifugation method. Ultracentrifugation and Invitrogen kit are methods suitable for subsequent observation of EV by TEM. EV isolated by the three methods can be detected by LFIA, being ExoQuick the one that provides higher signal intensities and requiring a minimal sample volume. In fact, this commercial kit was the method employed for EV enrichment in a pilot study in patients with chronic fatigue syndrome [35], according to our comparison report. Therefore, these results contribute to the current challenge of standardization of EV isolation protocols and pave the way for clinical applications with diagnostic or therapeutic objectives. 

## Figures and Tables

**Table 1:** Summary of the main aspects evaluated in our comparison study. * Not directly from isolated EV fractions, as previous protein precipitation or isolation without Proteinase K treatment is needed.

	UC	Invitrogen	ExoQuick
Starting volume	1 mL	250 µL	250 µL
Time requirements	~5–6 h	~1.5 h	~2 h
Protein [mg/mL]	3.05 ± 0.19	4.76 ± 2.09	27.98 ± 4.66
Suitable for SDS-PAGE/Western blot	Yes	No *	Yes
Suitable for dynamic light scattering (DLS) (mean diameter, nm)	Yes 152.09 ± 29.38	Yes 76.64±16.17	Yes 123.55 ± 63.04
Suitable for nanoparticle tracking analysis (NTA) (mean diameter, nm) [particles/mL]	Yes 208.5 ± 10.60 ~10^11^	Yes 203.67 ± 55.41 ~10^12^	Yes 233.97 ± 33.73 ~10^12^
Suitable for TEM	Yes	Yes	No
Suitable for lateral flow immunoassay (LFIA)	Yes	Yes	Yes

## Abbreviations

DLS: dynamic light scattering; EV: extracellular vesicles; LB: Laemmli buffer; LFIA: lateral flow immunoassay; NTA: nanoparticle tracking analysis; PFP: platelet-free plasma; TEM: transmission electron microscopy.
